# Modulating Linker Composition of Haptens Resulted in Improved Immunoassay for Histamine

**DOI:** 10.3390/biom9100597

**Published:** 2019-10-11

**Authors:** Lin Luo, Xiao-Qun Wei, Bao-Zhu Jia, Jin-Yi Yang, Yu-Dong Shen, Bruce Hammock, Jie-Xian Dong, Hong Wang, Hong-Tao Lei, Zhen-Lin Xu

**Affiliations:** 1 Guangdong Provincial Key Laboratory of Food Quality and Safety, South China Agricultural University, Guangzhou 510642, China; lin.luo@scau.edu.cn (L.L.); weixqun@scau.edu.cn (X.-Q.W.); yjy361@163.com (J.-Y.Y.); shenyudong@scau.edu.cn (Y.-D.S.); gzwhongd@163.com (H.W.); hongtao@scau.edu.cn (H.-T.L.); 2 College of Biology and Food Engineering, Guangdong University of Education, Guangzhou 510303, China; jiabaozhu@gdei.edu.cn; 3 Department of Entomology and Nematology and UCD Comprehensive Cancer Center, University of California Davis, Davis, CA 95616, USA; bdhammock@ucdavis.edu (B.H.); jxdong@ucdavis.edu (J.-X.D.)

**Keywords:** Histamine, Hapten design, monocloanl antibody, derivatization-free immunoassay

## Abstract

Histamine (HA) is an important food contaminant generated during food fermentation or spoilage. However, an immunoassay for direct (derivatization free) determination of HA has rarely been reported due to its small size to induce the desired antibodies by its current hapten-protein conjugates. In this work, despite violating the classical hapten design criteria which recommend introducing a linear aliphatic (phenyl free) linker into the immunizing hapten, a novel haptens, HA-245 designed and synthesized with a phenyl-contained linker, exhibited significantly enhanced immunological properties. Thus, a quality-improved monoclonal antibody (Mab) against HA was elicited by its hapten-carrier conjugates. Then, as the linear aliphatic linker contained haptens, Hapten B was used as linker-heterologous coating haptens to eliminate the recognition of linker antibodies. Indirect competitive ELISA (ic-ELISA) was developed with a 50% inhibition concentration (IC_50_) of 0.21 mg/L and a limit of detection (LOD) of 0.06 mg/L in buffer solution. The average recoveries of HA from spiked food samples for this ic-ELISA ranged from 84.1% and 108.5%, and the analysis results agreed well with those of referenced LC-MS/MS. This investigation not only realized derivatization-free immunoassay for HA, but also provided a valuable guidance for hapten design and development of immunoassay for small molecules.

## 1. Introduction

**Scheme 1:**
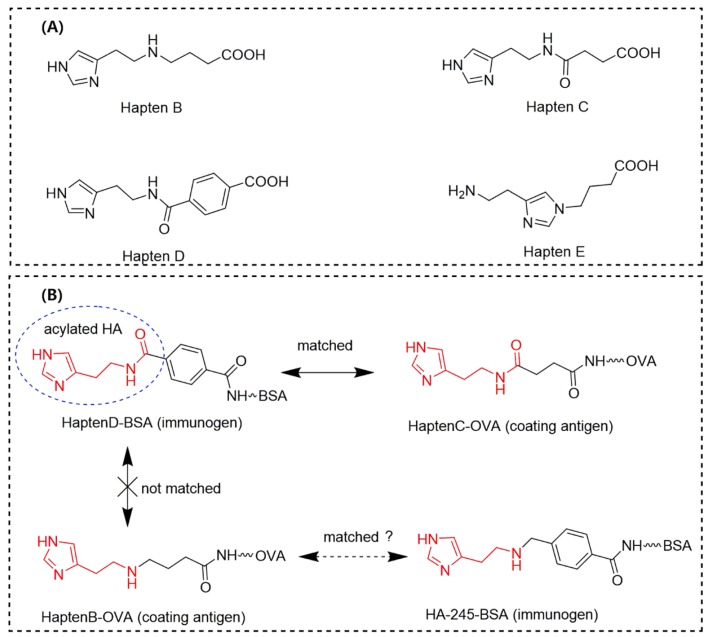
Structure of related histamine haptens (**A**); Schematic illustration of the hypothesis for the novel hapten design strategy against histamine (**B**).

Histamine (HA) is one of several biogenic amines closely associated with allergies and food poisonings. Fermented foods and deteriorated protein-rich foods are vulnerable to be contaminated by a high level of HA [1]. To ensure human health, many countries and communities including European Union (EU), United States Food and Drug Administration (FDA) and China, have set a permissible limit for HA in fish products in the range of 50 to 1000 mg/L or kg [2,3]. For effective supervision of HA residues in foods, it is indispensable to develop a rapid and reliable screening method for HA residues. While several immunoassays for HA have been developed, most of them were based on antibodies showing a high affinity to HA derivatives, such as succinylated HA [4], *p*-benzoquinone−histamine conjugate [5,6], chloroethyl-nitrosourea-histamine conjugate [7], nitrobenzoylated histamine (NPHA) [8], but negligible affinity to free HA. Furthermore, two immunoassays which could directly detect HA without sample derivatization were developed by Hammar et al [9] and Schneider et al [10], respectively. Hammar et al designed a HA hapten by attaching a linear aliphatic linker on the ring 1-nitrogen of HA, and conjugated it with dog serum albumin to form the immunogen. However, Hammar et al’s approach seems inappropriate, because the resulting monoclonal antibody exhibited a 10-fold higher affinity to 1-Methylhistamine, one of the main metabolites of HA, than to HA. In Schneider’s work, a specific anti-HA polyclonal antibody was elicited by an immunogen derived from the conjugation of HA with keyhole limpet hemocyanin via glutaraldehyde (as a linear aliphatic linker). However, the repeatability of Schneider’s strategy has been very poor since attempts using the same strategy to produce specific anti-HA antibodies have failed four times in our group. Besides, the authors further designed another two HA haptens by appending a linear aliphatic linker in primary amine group (Hapten B, Scheme 1A) and ring 1-nitrogen (Hapten E, Scheme 1A) of HA, respectively. However, the resulting antisera elicited by their hapten-protein conjugates still showed low titer and negligible binding affinity to HA [8]. 

In fact, sample derivatization should be avoided for most immunoassays to shorten the assay time and simplify the test procedure. Therefore, it is still of great significance to develop an antibody which could directly bind to HA with desirable affinity, thus developing derivatization-free immunoassay. Previously, the authors developed an ELISA for the indirect detection of HA [8]. In that work, a phenyl-contained derivative of HA (Hapten D, Scheme 1A) was employed as an immunizing hapten, and a polyclonal antibody which could recognize that nitrobenzoylated HA (NPHA) not intact HA was produced. During the development of ELISA for HA, Hapten B and Hapten C (Scheme 1A) were used as heterologous coating hapten to enhance assay sensitivity. It was found that the anti-NPHA antibody showed a high titer of 1:16,000 under Hapten C-OVA coating. This suggests that the anti-NPHA antibody also has a moderate affinity to acylated HA moiety. However, the anti-NPHA antibody shows no binding ability to Hapten B-OVA conjugate with the observed absorbance being at a blank level, suggesting the detrimental effect of modification of the amide (in Hapten C, Scheme 1B) to a secondary amine (in Hapten B, Scheme 1B). These results meant the incorporation of phenyl into immunizing hapten could significantly improve immunological properties of resultant anti-hapten immunogen. However, the benzoylation of HA strikingly changed the electronic properties of the primary amine nitrogen atom of HA, which might lead to the failure in recognizing the HA moiety in Hapten B by the anti-NPHA antibody. Therefore, it was queried whether maximizing the charge distribution similarity with intact HA by N-alkylation of HA with a phenyl-contained linker in its primary amine group (yield a secondary amine group) might elicit antibodies which could recognize HA with the desired affinity. Furthermore, the strong recognition of anti-NPHA antibody to acylated HA moiety also encouraged the hypothesis that, under the prerequisite of maximizing electronic and conformational similarity between hapten and target molecule, introducing a phenyl-contained linker into the immunizing hapten to improve immunological properties of anti-hapten immunogen, integrating with a heterologous coating strategy, might be a good choice for improving the immunoassay for small molecules. Herein, to investigate the effectiveness of the above hypothesis, hapten HA-245, which is hapten designed to have a phenyl-contained linker, was employed as immunizing haptens against HA. Meanwhile, Hapten B, a hapten containing a linear aliphatic linker, was used both as the control immunizing hapten and linker-heterologous coating hapten. 

## 2. Experimental

### 2.1. Molecular Modeling

The minimum energy geometries of haptens were optimized by a SKETCH module of SYBYL-X 2.0. The criteria of the termination were set at 0.005 kcal/(mol × Å) and maximum iterations were 1000. Then, the atom charge was calculated by the Gasteiger-Huckel method.

### 2.2. Hapten Synthesis

**Figure 1:**
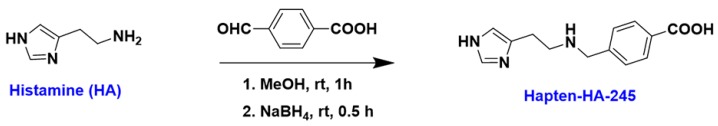
Structure and synthetic route of hapten for histamine.

Hapten B had been synthesized in our previous work [8]. The synthetic route of HA-245 (4-(((2-(1H-imidazol-4-yl)ethyl) amino) methyl) benzoic acid) was presented in Figure 1**.** Briefly, histamine dihydrochloride (1.84 g, 10 mmol) and sodium methoxide (1.08 g, 20 mmol) were dissolved in methanol, then 4-formylbenzoic acid (1.5 g, 10 mmol) was added to the resulting mixture under stirring. After 1 h, NaBH4 (0.37 g, 10 mmol) was added to the mixture, and the reaction proceeded another 0.5 h. Then, the solvent was removed under roto evaporation. The residue was purified by eluting with CHCl_3_/MeOH/NH_4_OH (10:5:1) through a silica gel column, and then crystallized from cold ethanol (10 mL) to afford Hapten-HA-245 (0.84 g, 3.4 mmol). The ESI analysis (positive ion) m/z 246 [M + H]^+^; ^1^H NMR (600 MHz, D_2_O) δ = 8.48 (d, *J* = 1.2, 1H), 7.72 (d, *J* = 8.1, 2H), 7.36 (d, *J* = 8.2, 2H), 7.22 (s, 1H), 4.20 (s, 2H), 3.32 (t, *J* = 7.6, 2H), 3.08 (t, *J* = 7.6, 2H). 

### 2.3. Conjugation of Haptens

As with Hapten B, HA-245 also has a secondary amine and a carboxyl group, thus HA-245 was conjugated to carriers (BSA or OVA) using the same one-step EDC method as that for preparation of Hapten B-protein conjugates instead of the two-step activated ester method to avoid lactamization. Briefly, 0.12 mmol of hapten and 67 mg of BSA (or 45 mg of OVA) were added in 10 mL of H_2_O. The resultant solution was cooled in an ice bath and the pH was adjusted to 4.5 with HCl (1M). Then, 320 mg of 1-(3-dimethylaminopropyl)-3-ethylcarbodiimide hydrochloride (EDC) was added (with stirring) to the above solution in small portions. The pH of this reaction mixture was kept at 4.5~5.0 (with HCl) throughout the addition of EDC and the mixture was then stirred at 4 °C over night. The conjugation mixture was dialyzed against 10 mM PBS (4 × 5 L) at 4 °C for 72 h, and diluted to 1.0 mg/mL with PBS and stored at –20 °C until used. 

### 2.4. Antibody Production

Animal manipulations were performed in compliance with the Regulation Guideline for Experimental Animals issued by the Ministry of Science and Technology of China (Ethical identification code: SYXK(Yue) 2014-0136; Approval date: 16 May 2017; Ethics committee: Guangdong Provincial Department of Science and Technology). 

Three Babl/c female mice aged 7 weeks were independently injected subcutaneously with 0.1 mg of immunogen (HA-245-BSA) in 0.2 mL of emulsion (a 1:1 mixture of immunogen (1 mg/mL) and complete Freund’s adjuvant). The booster immunizations were given 3 times using the same dose of immunogen emulsified in the incomplete Freund’s adjuvant were given at two-week intervals. One week after the last immunization, the mice were tail-bled and the blood samples could coagulate at 37 ℃ for three hours. Then, the antisera (polyclonal antibody) was separated (4000× *g*, 30 min) by centrifugation. The obtained antisera were tested for their anti-hapten antibody titer and binding properties by non-competitive indirect ELISA and ic-ELISA (the detailed procedure is presented in ELISA Protocol of Supplementary Materials). 

The mouse whose antisera showed the best binding ability to HA was chosen as the donor of spleen cells for cell fusion. The selected mouse received a final intraperitoneal injection with the same amount of immunogen in PBS. Three days later, the mouse was exsanguinated for cell fusion. Then, the obtained spleen cells were fused with SP2/0 murine myeloma cells to form hybridomas using PEG 2000 by the same procedure as described by Kane and Banks [11]. At 8 to 10 days after the cell fusion, when the hybridoma cells were grown to approximately 30–40% confluent in the well, culture supernatants were collected and screened using indirect ELISA for the presence of anti-HA antibodies. The hybridomas showing the desired specificity were sub-cloned for multiple rounds by the limiting dilution method until a pure and stable antibody-producing clone was obtained. The positive clones were injected into female Balb/c mice to obtain ascitic fluid for antibody production. The anti-HA Mab was prepared through ascites production and purified by ammonium sulfate precipitation followed by a protein-G column. The obtained anti-HA Mab was stored at –20 °C until used.

### 2.5. ELISA Protocol

The ELISA was constructed based on the regular procedure of competitive indirect ELISA (ic-ELISA) [12]. For further details, see the Supplementary Materials. 

### 2.6. Cross-Reactivity Test

The specificity of the antibody was assessed by testing cross-reactivities (CRs) with some compounds (cross-reactants) which are structurally or functionally related with target molecule using ic-ELISA. The CR was calculated using the following equation:

CR% = IC_50_ (Histamine)/IC_50_ (cross-reactants) ×100 (1)

## 3. Results 

### 3.1. Characterization of Hapten and Artificial Antigens

**Figure 2:**
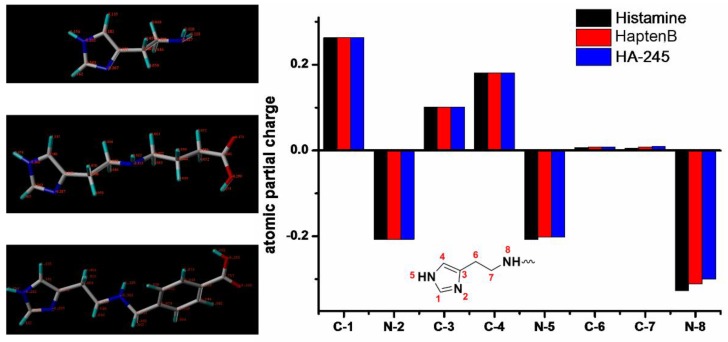
Computed partial atomic charges of histamine (HA), Hapten B and hapten HA-245. Only those atoms that are shared by these structures are displayed. Hydrogen atoms are not included.

Hapten HA-245 designed with a phenyl-contained linker was synthesized by condensation of HA with 4-formyl-benzoic acid, followed by reduction of schiff base (C-N double bond) to secondary amine by NaBH_4_ (Figure 1). Hapten B, a HA hapten having a linear aliphatic linker, had been prepared in our previous work [8]. The ESI-MS and NMR test results indicated the success of hapten synthesis (Figures S1 and S2). It was shown in Figure 2 that the HA moiety was well retained in HA-245, and the phenyl in the linker had a negligible effect on the electronic distribution of HA since only a slight alteration (Gasteiger−Huckel charge change from −3.27 to −3.00) on N8 atom was observed, implying that the introduction of phenyl-contained linker induced negligible interference over the electronic nature of the target moiety as well. HA-245 was conjugated to BSA and OVA as immunogen and coating antigen, respectively. The resulting hapten-BSA conjugates were of comparable hapten density, with the hapten to protein molar ratio in the range of 18.3~23.4, as determined by Habeeb’s TNBS method [13].

### 3.2. Production of Monoclonal Antibody

**Figure 3:**
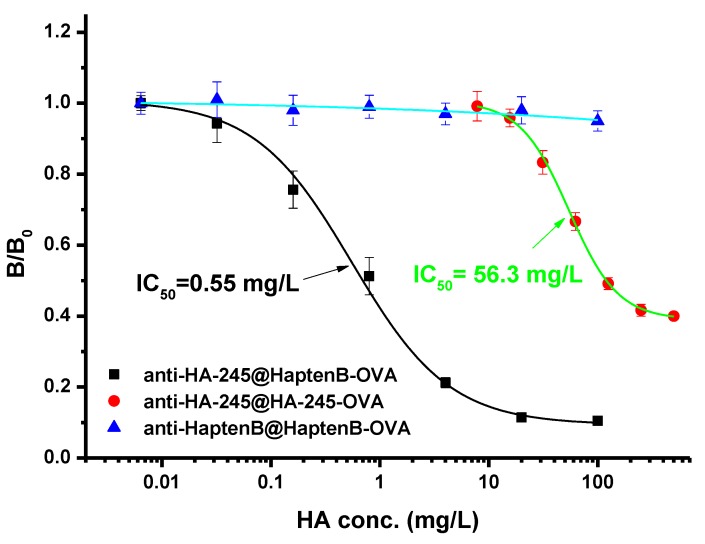
ic-ELISA inhibition curves of anti-HA-245#2 and anti-Hapten B#1 using Hapten B-OVA and HA-245-OVA as coating antigen, respectively.

The efficacy of two immunogens (HA-245-BSA, Hapten B-BSA) were assessed by immunization of groups of n = 3 Balb/c mice. Each immunogen was administered on weeks 0, 2, 4 and 6 with bleeds taken one week following the last immunization. The anti-HA antiserum titers and binding properties were determined by ELISA using Hapten B-OVA and HA-245-OVA as coating antigen, respectively. As shown in Table 1, the mice antiserum elicited by Hapten B-BSA were of low titer and no binding ability to HA, with the highest titer of 1:4000 and no inhibition even under high level of HA (50 mg/L), paralleling results with these previously observed for immunization of New Zealand white rabbits [8]. Gratifyingly, the antiserum against HA-245 were of remarkable enhanced titer with titer of 1:16,000~1:32,000 and 1:128,000~1:256,000 under Hapten B-OVA coating (heterologous coating) and HA-245-OVA coating (homologous coating), respectively. Meanwhile, their binding capability with free HA was observed. As shown in Table 1, anti-HA-245#2 exhibited a moderate inhibition of 37.5% in the presence of 50 mg/L of HA under homologous coating, but the inhibition increased significantly to 95.1% when using heterologous coating antigen to eliminate the recognition of the antibody to the linker. The inhibition curves of anti-HA-245#2 and anti-HaptenB#1 were constructed using HA-245-OVA and Hapten B-OVA as the coating antigen, respectively. However, no absorbance was observed for the anti-HaptenB#1@HA-245-OVA antibody@coating antigen combination, thus the corresponding inhibition curve missed. Figure 3 showed that the anti-HA-245#2@ Hapten B-OVA heterologous combination exhibited the highest sensitivity for HA with an IC_50_ of 0.55 mg/L which was improved ~100-fold compared to that of the anti-HA-245#2@HA-245-OVA homologous combination. Therefore, the hapten design strategy proposed above proves to be effective for HA. Furthermore, the NO.2 mice immunized by immunogen HA-245-BSA was chosen as the donor of the spleen cells for cell fusion. It was found that five wells among 480 wells were determined to have positive hybridoma cells which were subcloned five times using the limiting dilution method. One hybridoma (3E9) secreting Mab showing affinity to the HA was obtained. 

### 3.3. Development of ic-ELISA for Histamine

**Figure 4:**
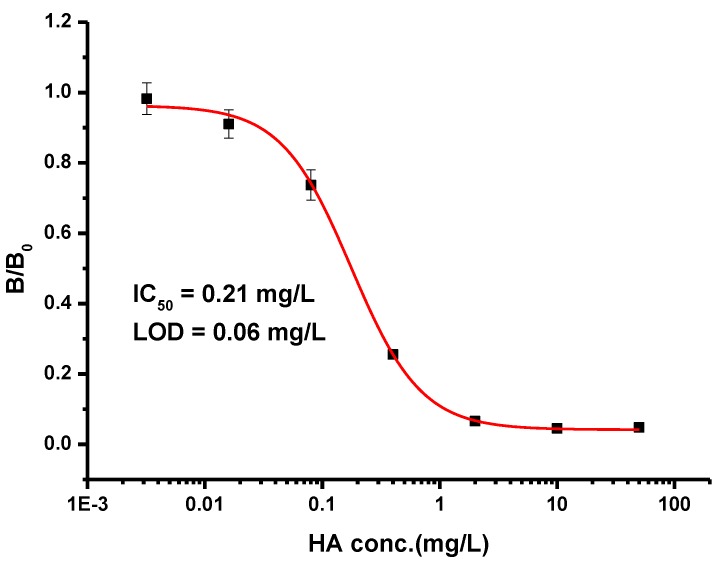
Anti-HA Mab based ic-ELISA calibration curve for HA (*n* = 3).

The obtained anti-HA Mab was further used to develop an ic-ELISA for HA. To achieve optimal assay performance, some key parameters including the amount of antibody and coating antigen, pH and ionic strength of the assay buffer were optimized. Under each condition, ic-ELISA inhibition curves for HA were developed, and then several index including Amax (the absorbance value of negative control), IC_50_ (the HA dose that produce 50% decrease in Amax) and ratio of Amax to IC_50_ (Amax/IC_50_) were compared. The highest ratio of Amax to IC_50_ indicated the optimal condition. As shown in Table S1, the optimal coating antigen concentration and antibody dilution was at 500 ng/mL and 1:16,000, respectively. PBS with series of PO_4_^3–^ strength (5–40 mmol/L), and pH values (5.6–8.5) were used as a working buffer. As a result, PBS with a PO_4_^3–^ strength of 20 mmol/L and a pH of 6.8 were confirmed to be the optimal working buffer. Under the optimal condition, the standard ic-ELISA inhibition curve for HA was constructed (Figure 4). The IC_50_ value and LOD (IC_10_) were 0.21 mg/L and 0.06 mg/L, respectively. 

The cross-reactivity (CR) test was carried out by measuring CRs between anti-HA Mab and several HA or hapten HA-245 related compounds through ic-ELISA. It was shown in Table 2 that this Mab exhibited negligible CRs (<0.1%) with HA structurally related biogenic amines or L-histidine, the precursor of HA. In contrast to the antibody in Hammar’s work [9], anti-HA Mab could not recognize 1-Methylhistamine, with the CR below 0.1%. This phenomenon might be due to that the linker in hapten HA-245 was attached on the side chain primary amine group of HA. Thus, imidazole ring moiety is kept in a position distal from carrier protein, which made it to be the potential immunodominant moiety for antibody formation. For 1-Methylhistamine, the methylation in imidazole ring 1-nitrogen remarkably changed the geometry and charge distribution of imidazole ring, which would make the anti-HA Mab not recognize it. Although the anti-HA Mab showed the highest affinity to immunizing hapten (HA-245), with a CR of 39,583.3%, HA-245 is a novel compound first synthesized in this work, and does not occur naturally. Thus, it would not interfere with the HA analysis by this ic-ELISA. In addition, the anti-HA-245#2 showed negligible CRs with 4-(aminomethyl)-benzoic acid and benzoic acid which are similar to the linker moiety in hapten HA-245. These results suggested that ic-ELISA constructed on anti-HA Mab @ Hapten B-OVA combination is of good specificity to HA.

The high levels of HA usually contaminate seafood, especially, Scombroid fish such as tuna, mackerel, bonito, and saury that are rich in free histidine in their muscle [14,15]. Meanwhile, the HA content in fermented foods, such as wine, soy sauce, and yoghurt might also significantly increase due to microbial action [16,17,18]. Therefore, saury, red wine, soy sauce and yoghurt samples were chosen as model sample matrices to assess the reliability of the ic-ELISA for real samples analysis. Initially, the sample matrix effect should be evaluated and overcome. The spiked samples were extracted, diluted 5-fold, 10-fold, 20-fold and 40-fold with PBS (10 mM, pH 7.4), respectively, and then analyzed by ic-ELISA. As shown in Figure S3, the interferences from the sample matrix gradually bated as the dilution increased; and satisfactory recoveries for HA fortified food samples (saury fish: 85.6%; red wine: 88.3%; soy sauce: 95.2% for spiked; yoghurt 93.1%) were achieved, when the fortified saury fish sample extract, fortified red wine sample, fortified soy sauce sample extract and fortified yoghurt sample extract were diluted 20-fold, 10-fold, 10-fold and 10-fold, respectively. Therefore, a dilution factor of 20-fold for saury samples and 10-fold for red wine, soy sauce and yoghurt samples were adopted during sample preparation. As a result, the sample preparation procedures were as follow: 2 g of saury fish sample was mixed with 2 mL of distilled water and homogenized, and then 2 g of the homogenate was added to 18 mL of PBS (10 mM, pH 7.4), and vibrated for 1 min. The mixture was centrifuged at 3000× *g* for 10 min, after removing the fat layer, the supernatant was ready for ic-ELISA analysis. Then, 1 mL of red wine or soy sauce was diluted 10-fold in PBS (10 mM, pH 7.4) and subjected to the ic-ELISA analysis. Further, 1 g of yoghourt and 9 mL of PBS (10 mM, pH 7.4) were mixed and vibrated for 1 min. The mixture was centrifuged at 3000× *g* for 10 min, the supernatant was ready for ic-ELISA analysis.

The recovery tests for the intra-assay and inter-assay were performed by analyzing saury, red wine, soy sauce and yoghurt samples spiked with four levels of HA (0, 2.0, 5.0 and 10.0 mg/L or kg). As shown in Table 3, the average recoveries for intra-assay were from 87.5% to 106.8%, with the coefficient of variation below 15.0%. For the inter-assay, the average recoveries were between 84.1% and 108.5%, with the CV ranging from 7.9% to 14.0%. These results of intra-assay and inter-assay indicated that this ic-ELISA has good accuracy and precision.

Sixteen blind samples were purchased from local supermarkets, then their HA contents were analyzed by both this ic-ELISA and LC-MS/MS which was established in our previous work [8], respectively. As shown in Table S2, the results of the ic-ELISA correlated well with those of LC-MS/MS, suggesting the good accuracy and reliability of the ic-ELISA for detection of HA in real samples.

## 4. Discussion

The incorporation of a linker ending with a reactive group when designing hapten was generally preferred. According to conventional hapten design criteria, a linear aliphatic linker with a length of 2 to 6 carbon atoms was preferable for an optimal hapten, since it caused minimum physico-chemical and antigenic interference over the hapten. To produce the desired antibodies, much more attention has been invested on the effect of the hapten linker site on the binding properties (affinity and specificity) of resultant antibodies, since the linker site would mold the final conformation of the conjugate and thus settle the specific moieties that could be accessible for binding. For example, Mercader et al. produced a high-affinity anti-pyraclostrobin antibody by synthesizing a series of anti-pyraclostrobin haptens with the same aliphatic linkers located at different sites, and found that the lower titers and affinities of one set of antibodies were most likely due to the conformational effects of the linker on the immunizing bioconjugate [19]. Wang et al. found that four linker sites on R-(−)-salbutamol modulating the class specificity of the resultant antibodies against 31 β-Agonists [20]. However, for smaller molecules like HA, acrylamide, ethyl carbamate and 3-amino-2-oxazolidinone (AOZ) with simpler structure and limited site for tethering the linker, shifting the linker site exerted negligible effect on the quality of the resultant antibodies, since almost all of the resulting antibodies against conventionally designed haptens exhibited low titer and negligible affinity for target molecules (as summarized in Table S3). These results suggested that these conventionally designed haptens failed to evoke significant anti-hapten immune response. It is generally agreed that a weak interaction of T cell receptor (TCRs) with antigenic molecules bound and presented by the major histocompatibility complex (MHC) may result in the failure in evoking a significant immune response [21]. Despite introducing a linear aliphatic linker, these conventionally designed haptens are still of diminutive size and low hydrophobicity which are adverse factors for T cell receptor binding [22]. In contrast, the remarkably enhanced quality of antibodies elicited by hapten HA-245 sharing a phenyl-contained linker indicated that the phenyl contributed significantly to the markedly improved immune response against HA. This could be attributed to the increased size and hydrophobicity in hapten resulting from the incorporation of phenyl, thus boosting TCR binding and overcoming the poor immunogenicity of native hapten.

Nonetheless, it should be noted that the incorporation of phenyl-contained linker should induce a minimum possible alteration in the electronic and conformational properties of the target molecules. As seen with the anti-NPHA hapten (Hapten D, Scheme 1A) mentioned above, it is a phenyl-contained derivative synthesized by benzoylation of HA with a phenyl-contained linker in the primary amine of HA. However, the resulting antibodies could not recognize the intact HA but acylated HA moiety, which is likely due to the significant change of charge distribution on N8 atom of HA (Gasteiger−Huckel charge change from −3.27 to −2.84) resulting from the modification of primary amine to amide. Nevertheless, hapten HA-245 synthesized via N-alkylation of HA with a phenyl-contained linker in its primary amine group with a slight modification of a charge distribution on N8 atom, elicited antibodies which could recognize intact HA with desired affinity. 

As guided by classical hapten design criteria, phenyl should not be incorporated into linker because it is easy to elicit linker antibodies and thereby induce linker recognition. Actually, in this work, all resulting antibodies elicited by hapten HA-245 sharing a phenyl-contained linker exhibited obvious linker recognition, and their binding abilities with HA were masked to a large extent when performing ic-ELISA using homogenous coating haptens. However, the linker recognition was eliminated in heterologous ic-ELISA where hapten (Hapten B) designed to have an aliphatic linker in the same position as that in immunizing hapten (HA-245) was used as linker-heterologous coating hapten, and significantly improved the binding abilities with HA were observed for these antibodies.

In summary, this investigation demonstrated the effectiveness of modulating the linker composition of immunizing haptens from conventionally preferred linear aliphatic linker to a phenyl-contained linker in improving the quality of antibodies against HA. However, for this hapten design strategy, it should be noted: (i) The incorporation of phenyl-contained linker should not induce physico-chemical and antigenic interference over the hapten as much as possible, which could be assessed by the computer-assisted molecular modeling; (ii) when developing the immunoassay, it is necessary to eliminate the linker recognition by using linker-heterologous haptens which have a phenyl-free linker as competitive haptens. This work realized derivatization-free immunoassay for HA and provided a valuable guidance for hapten design and development of immunoassays for other small molecules.

## Figures and Tables

**Table 1:** Characterization of anti-HA serum obtained after the fourth immunization by competitive and non-competitive ELISA.

	Coating Antigen			
Antibody	Hapten B-OVA		HA-245-OVA	
	Titer^b^ (×10^3^)	Inhibition^c^ (%)	Titer (×10^3^)	Inhibition (%)
anti-Hapten B#1	4	—	<1	—
anti-Hapten B#2	2	—	<1	—
anti-Hapten B#3	4	—	<1	—
anti-HA-245#1^a^	16	89.8	128	26.6
anti-HA-245#2	16	95.1	128	37.5
anti-HA-245#3	32	92.3	256	31.8

^a^ anti-HA-245#1 represents the antiserum from NO.1 mice immunized by immunogen derived from hapten HA-245. ^b^ Titer is defined as dilution factor of antiserum with the absorbance at 450 nm being situated at about 1.0 at coating concentration of 1.0 mg/L. ^c^ Percentage inhibition was expressed as follow: inhibition (%) = [1 − (B/B_0_)] × 100. B_0_ was mean absorbance of the wells in the absence of HA; B was mean absorbance of the wells in the presence of 50 mg/L of HA.

**Table 2:** The cross-reactivity of anti-HA Mab with HA and related compounds.

Compound	Structure	IC_50_ (mg/L)	CR (%)
HA	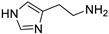	0.21	100
Hapten-HA-245	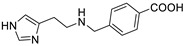	0.0012	39,583.3
L-histidine	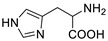	>5000	<0.1
1-Methyl-histamine	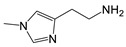	>5000	<0.1
Tryptophan	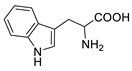	>5000	<0.1
Tryptamine	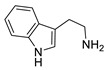	>5000	<0.1
Tyramine	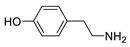	>5000	<0.1
Phenethylamine	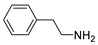	>5000	<0.1
4-(aminomethyl)-benzoic acid	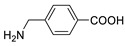	>5000	<0.1
Benzoic acid	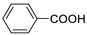	>5000	<0.1

**Table 3:** Recoveries of histamine from spiked food samples by ic-ELISA (*n* = 3).

Sample	Spiked (mg /kg or mg/L)	Intra-Assay ^a^			Inter-Assay ^b^		
		Measured (mg /kg or mg/L)	Recovery (%)	CV (%)	Measured (mg /kg or mg/L)	Recovery (%)	CV (%)
Saury	0	2.31	—	13.0	2.40	—	12.5
	2.0	4.05	87.5	15.0	4.32	96.0	14.0
	5.0	6.72	88.4	10.4	7.27	97.4	11.1
	10.0	11.25	89.5	11.6	10.81	84.1	13.0
Red Wine	0	0.91	—	11.1	1.13	—	10.7
	2.0	2.80	95.0	10.7	2.95	92.5	10.3
	5.0	6.44	106.8	7.8	6.36	105.2	7.9
	10.0	10.58	96.8	10.5	11.22	101.2	11.6
Soy Sauce	0	1.22	—	12.6	1.30	—	11.8
	2.0	3.08	93.0	10.5	3.28	99.0	10.5
	5.0	6.35	102.6	13.2	6.24	98.8	12.1
	10.0	10.1	88.8	12.1	9.82	85.2	13.2
Yoghurt	0	ND ^c^	—	—	ND ^c^	—	—
	2.0	1.92	96.0	10.2	2.16	108.5	12.6
	5.0	5.24	104.8	11.4	4.96	99.2	10.8
	10.0	9.76	97.6	13.6	10.24	102.4	11.8

^a^ intra-assay variation was determined by three replicates on a single day. ^b^ inter-assay variation was determined by three replicates on three consecutive days. ^c^ ND, not detected. Data were below the LOD of the assay in corresponding sample.

## Supplementary Materials

The following are available online at https://www.mdpi.com/2218-273X/9/10/597/s1, Figure S1: ^1^H NMR spectrum of hapten HA-245, Figure S2: ESI-MS spectrum of hapten HA-245, Figure S3: Effect of different matrix dilution factors on recoveries of HA from the saury, red wine, soy sauce and yoghurt samples spiked at 5 mg/kg or 5 mg/L (*n* = 3), Table S1: Effect of physicochemical parameters on ic-ELISA performance (*n* = 3), Table S2: Comparison of the blind analysis results for HA by ic-ELISA and LC-MS/MS, Table S3: Synopsis of conventional linear aliphatic linker contained haptens against histamine, acrylamide, ethyl carbamate and AOZ.
