# Supplementary material for: Modulating Linker Composition of Haptens Resulted in Improved Immunoassay for Histamine
Source: Biomolecules. 2019 Oct 11;9(10):597. doi: 10.3390/biom9100597 (PMC6843301; doi:10.3390/biom9100597)
Supplement: Supplementary file 1 [file biomolecules-09-00597-s001.pdf]

Supplementary Materials

Modulating Linker Composition of Haptens Resulted in Improved Immunoassay for Histamine

Lin Luo[a], Xiao-Qun Wei[a], Bao-Zhu Jia[b], Jin-Yi Yang[a], Yu-Dong Shen[a], Bruce Hammock[c], Jie-Xian Dong[c], Hong Wang[a], Hong-Tao Lei[a], Zhen-Lin Xu[a,*]

[a] Guangdong Provincial Key Laboratory of Food Quality and Safety, South China Agricultural University, Guangzhou 510642, China

[b] College of Biology and Food Engineering, Guangdong University of Education, Guangzhou 510303, China

[c] Department of Entomology and Nematology and UCD Comprehensive Cancer Center, University of California Davis, Davis, CA 95616, USA

Contents:

1. Animals and Reagents.

2. Instruments.

3. ELISA Protocol

4. Figure S1~S3, Table S1, Table S2 and Table S3

9. References

1. Animals and Reagents. Histamine dihydrochloride, L-histidine, L-tryptophan, tryptamine hydrochloride, tyramine hydrochloride, phenethylamine hydrochloride, cadaverine hydrochloride, 4-(2-aminoethyl) benzoic acid, 4-(aminomethyl) benzoic acid, 4-aminobutyric acid, acryloyl chloride, diethyl dicarbonate (DEPC) were obtained from Heowns Biochem Technologies Co. Ltd. (Tianjin, China). sodium borohydride ($NaBH_4$), dimethylformamide (DMF), 1-(3-Dimethylaminopropyl)-3-ethyl-carbodiimide hydrochloride (EDC) and triethylamine were obtained from Aladdin Chemical Technology Co., Ltd. (Shanghai, China). Histamine, 3,3',5,5'-tetramethylbenzidine (TMB), glutaraldehyde (50%, v/v), complete and incomplete Freund's adjuvants, Keyhole Limpet Hemocyanin (KLH), bovine serum albumin (BSA), ovalbumin (OVA), horseradish peroxidase and peroxidase-labelled goat anti-rabbit IgG (secondary antibody) were obtained from Sigma-Aldrich (St. Louis, MO, USA). *N, N*-Dimethylformamide (DMF), acetonitrile (ACN), Tween-20, ethanol, methanol (MeOH), ethyl acetate (EtOAc), tetrahydrofuran (THF) and chloroform ($CHCl_3$) were obtained from Damao Chemical Reagent Co., Ltd. (Tianjin, China). BABL/c female mice aged 7 weeks were supplied by the Guangdong Medical Experimental Animal Centre. 96-well polystyrene micro-plates were obtained from Shenzhen Jinchanhua Industrial Co. Ltd. (Shenzhen, China). All other reagents were of analytical reagent grade or higher purity.

2. Instruments. ELISA Plates were washed in a Multiskan MK2 microplate washer (Thermo Scientific, Hudson, NH, USA). Absorbance was measured at a wavelength of 450 nm using a Multiskan MK3 microplate reader (Thermo Scientific). Ultraviolet

spectrometry (UV) was recorded on a UV-3010 spectrophotometer (Hitachi, Tokyo, Japan). LC–MS/MS analysis was carried out by using the 1200 series LC system (Agilent Technologies) equipped with the Agilent 6410 Triple Quad LC–MS System (Agilent Technologies). Nuclear magnetic resonance (NMR) spectra were achieved with either a DRX-400 or DRX-600 NMR spectrometer (Bruker, Rheinstetten, Germany).

3. ELISA protocol

The coating antigens (100 μL/well) diluted in carbonate buffer (50 mmol/L, pH 9.6) were added into microplates and incubated overnight at 37 °C. The microplates were then washed twice, and the blocking solution (200 μL/well) was added to block the unbound active sites at 37 °C for 3 h, and dried at 37 °C for 1 h. 50 μL of target analyte standards or diluted sample solutions and 50 μL of diluted anti-hapten antibodies in PBST were added into each well and incubated at 37 °C for 40 min. and then the wells were washed five times. 100 μL of HRP labeled secondary antibody (diluted 1:5000 in PBST) was then added to the each well and incubated for 40 min at 37 °C. The wells were washed again for five times before the addition of 100 μL of TMB-based substrate solution. The enzymatic chromogenic reaction proceeds for 12 min and was stopped by adding 50 μL of 2 M H_2SO_4. Finally, absorbance at 450 nm was recorded. The percentage inhibition percentage used to characterize the binding ability of antibodies to target molecule was calculated using the following formula: inhibition (%) = $[1 - (B/B_0)] \times 100$; Where B_0 represented the mean absorbance of the wells free from competitor (target analyte) ; B represented the mean absorbance of wells in the presence

of a certain level of competitor. Plotting B/B_0 (Y axis) against the logarithm of the target analyte concentration (X axis) yielded inhibition curve. A four-parameter logistic equation was adopted to fit the sigmoidal curve using Origin 8.6 software (Origin Lab Corp., Northampton, MA): Y = (A − D)/[1 + (x/C)B] + D, where A is the maximum response at high asymptotes of the curve, D is the minimum response at low asymptotes of the curve, C is the concentration of the analyte that lead to 50% inhibition, and E is the slope of sigmoidal curve. The limit of detection (LOD) was defined as the concentration of analyte that produce 10% inhibition (IC_{10}).

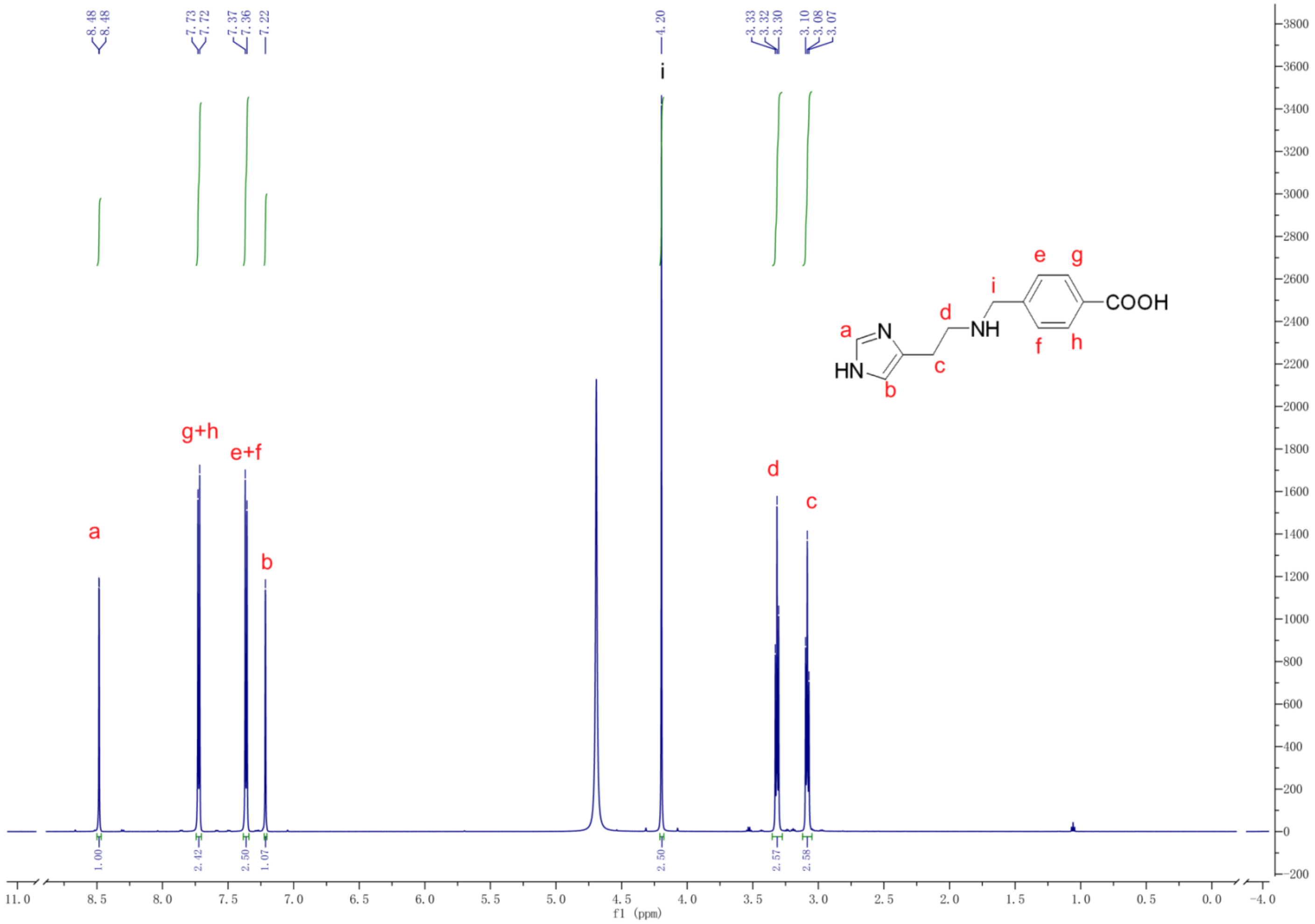

Figure S1.¹H NMR spectrum of hapten HA-245

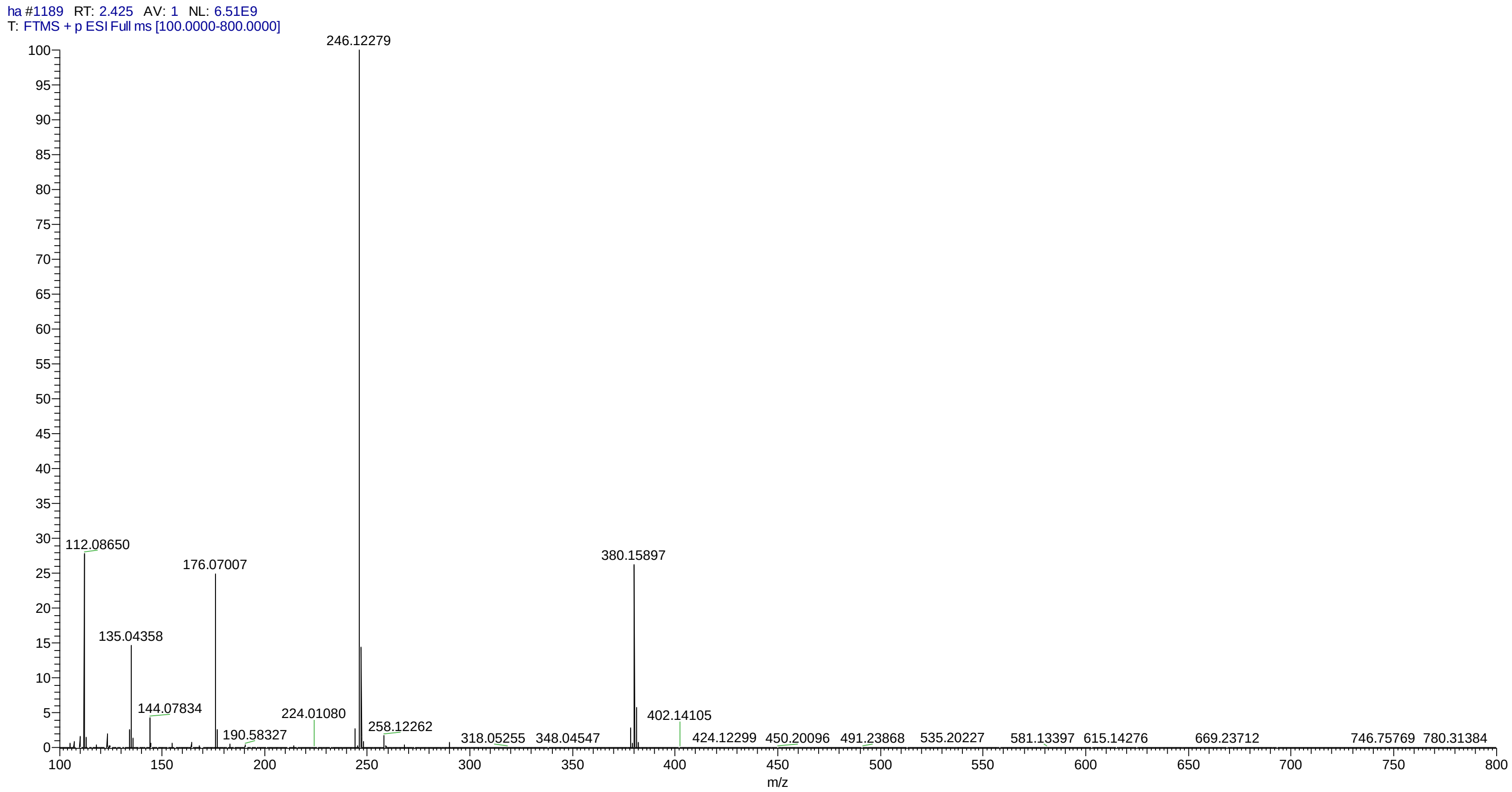

Figure S2. ESI-MS spectrum of hapten HA-245 (positive ion mode)

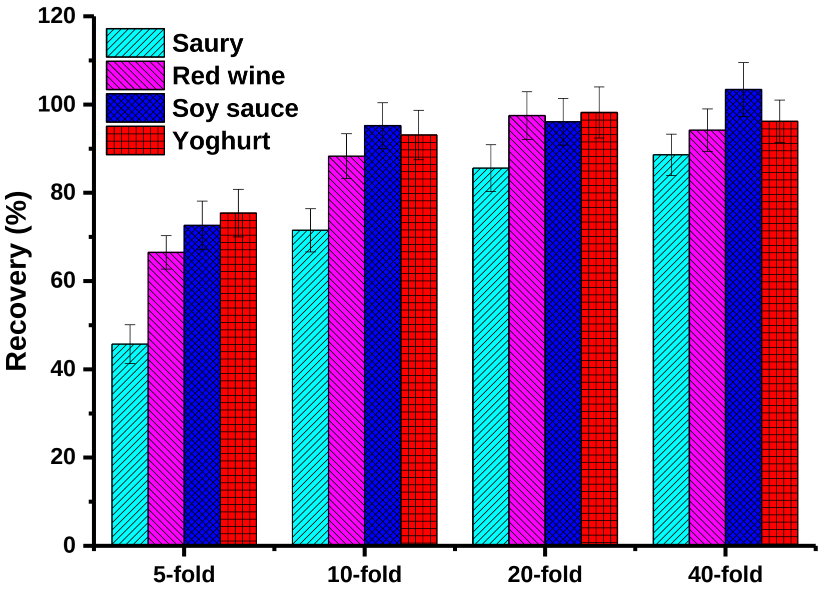

Figure S3. Effect of different matrix dilution factors on recoveries of HA from the saury, red wine, soy sauce and yoghurt samples spiked at 5 mg/kg or 5 mg/L (n=3).

Table S1. Effect of physicochemical parameters on ic-ELISA performance (n = 3).

Parameters	Amax	IC_{50} (mg/L)	Amax/IC_{50}
Coating concentration (ng/mL)/ antibody dilution			
2000/1:64000	1.14 ± 0.018	0.56 ± 0.08	2.04
1000/1:32000	1.37 ± 0.027	0.44 ± 0.07	3.11
500/1:16000	1.25 ± 0.022	0.34 ± 0.07	3.68
250/1:8000	1.03 ± 0.015	0.42 ± 0.06	2.45
PO_4^{3-} concentration (mmol/L)			
5	1.44 ± 0.021	0.52 ± 0.09	2.77
10	1.42 ± 0.030	0.45 ± 0.07	3.16
20	1.22 ± 0.019	0.32 ± 0.06	3.81
40	0.96 ± 0.017	0.54 ± 0.07	1.78
pH value			
5.6	1.02 ± 0.017	0.44 ± 0.06	2.32
6.2	1.13 ± 0.022	0.31 ± 0.05	3.65
6.8	1.17 ± 0.019	0.23 ± 0.06	5.09
7.4	1.09 ± 0.020	0.34 ± 0.07	3.21
8.5	1.01 ± 0.022	0.55 ± 0.08	1.83

Table S2. Comparison of the blind analysis results for HA by ic-ELISA and LC-MS/MS (n=3)[a].

sample	ic-ELISA (mg/L or mg/kg)	LC-MS/MS (mg/L or mg/kg)
saury#1[b]	7.82	7.24
saury#2	2.43	2.13
saury#3	12.06	11.4
saury#4	5.21	4.84
red wine#1	2.73	2.41
red wine#2	1.34	1.52
red wine#3	ND[c]	0.33
red wine#4	3.21	3.52
soy sauce#1	2.58	2.41
soy sauce#2	4.07	4.37
soy sauce#3	2.34	2.08
soy sauce#4	1.82	1.71
yoghurt#1	0.91	0.84
yoghurt#2	ND	0.52
yoghurt#3	ND	0.18
yoghurt#4	ND	0.32

[a]each sample was determined by ELISA and LC−MS/MS three times, respectively.

[b]saury#1 represents the NO.1 saury sample.

[c]ND, not detected. Data were below the LOD of the assay in corresponding sample.

Table S3**.** Synopsis of conventional linear aliphatic linker contained haptens against histamine, acrylamide, ethyl carbamate and AOZ

Target	Immunizing hapten	Immunogen	Quality of resulting antibodies	Reference
	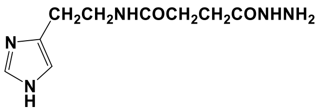	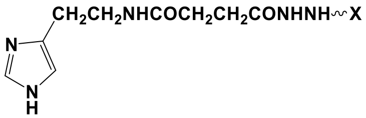	could not recognize the hapten moiety	Mita et al, 1984
	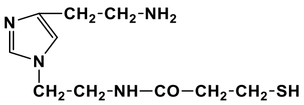	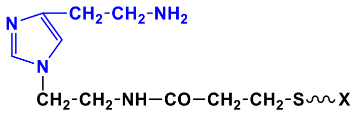	Affinity constant for HA: 1.0 × 10^7 L mol^{-1} Affinity constant for 1-Methyl-HA: 1.0 × 10^8 L mol^{-1}	Hammar et al, 1990
Histamine (Mw:111 Da)	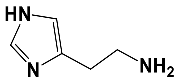	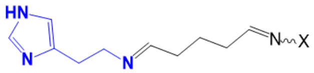	IC_{50}: 520 ng/mL	Schneider et al, 1996
			Titer: 1:4000; and no binding ability with free histamine were observed	Luo et al, 2014
	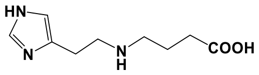 Hapten B	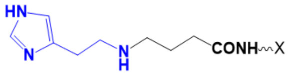	Titer: 1:4000; and no binding ability with free histamine were observed by ic-ELISA	Luo et al, 2014
	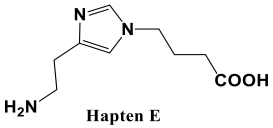 Hapten E	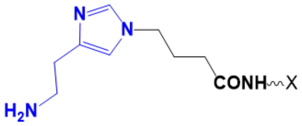	Titer: 1:4000; and no binding ability with free histamine were observed by ic-ELISA	Luo et al, 2014

Target	Immunizing hapten	Immunogen	Quality of resulting antibodies	Reference
Acrylamide (Mw: 71 Da)	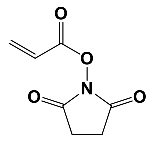 NAS	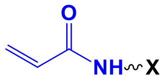	Affinity constant: 6.7 × 10^7 L mol^{-1}<br>Detection limit of BA-ELISA: 6 ng/mL	Zhou et al, 2008
			Titer: 1:8000<br>no binding ability with free acrylamide were observed by ic-ELISA	Wu et al, 2014
	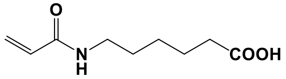	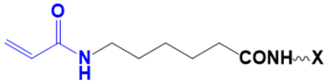	no significant binding to free acrylamide	Preston et al, 2008
	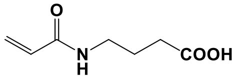	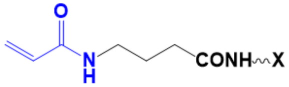	Titer: 1:4000<br>no binding ability with free acrylamide were observed by ic-ELISA	Wu et al, 2014
	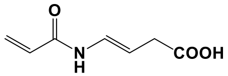	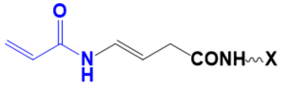	Titer: 1:256000<br>no binding ability with free acrylamide were observed by ic-ELISA	
Ethyl carbamate (Mw: 89 Da)	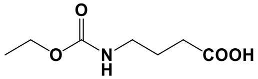	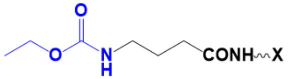	Titer: 1:4000<br>no binding ability with free ethyl carbamate were observed by ic-ELISA	Luo et al, 2017
AOZ (Mw:102 Da)	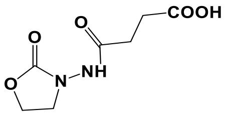	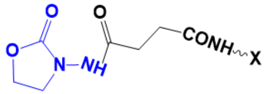	no binding ability with free AOZ were observed by ic-ELISA	Diblikova et al, 2006

Target	Immunizing hapten	Immunogen	Quality of resulting antibodies	Reference
AOZ<br>(Mw:102 Da)	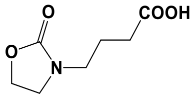	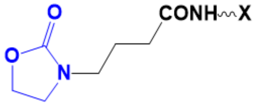	no binding ability with free AOZ were observed by ic-ELISA	Diblikova et al, 2006

X represents the carrier if any

References

Mita, H.; Yasueda, H.; Shida, T.; Baba, S., An attempt to produce an antibody of histamine and histamine derivatives. Agents and actions 1984, 14 (5-6), 574-579.

Hammar, E.; Berglund, A.; Hedin, A.; Norrman, A.; Rustas, K.; Ytterstrom, U.; Akerblom, E., An immunoassay for histamine based on monoclonal antibodies. Journal of immunological methods 1990, 128 (1), 51-58.

Schneider, E. U., E.; Martlbauer, E., Production and Characterization of Antibodies Against Histamine. In In Immunoassays for Residue Analysis: Food Safety;, Beier, R. C., Stanker, L. H., , Ed. ACS Symposium Series 621: American Chemical Society: Washington, DC,, 1996; pp 413-420.

Luo, L.; Xu, Z.-L.; Yang, J.-Y.; Xiao, Z.-L.; Li, Y.-J.; Beier, R. C.; Sun, Y.-M.; Lei, H.-T.; Wang, H.; Shen, Y.-D., Synthesis of Novel Haptens and Development of an Enzyme-Linked Immunosorbent Assay for Quantification of Histamine in Foods. Journal of Agricultural and Food Chemistry 2014, 62 (51), 12299-12308.

Zhou, S.; Zhang, C.; Wang, D.; Zhao, M., Antigen synthetic strategy and immunoassay development for detection of acrylamide in foods. Analyst 2008, 133 (7), 903-909.

Preston, A.; Fodey, T.; Elliott, C., Development of a high-throughput enzyme-linked immunosorbent assay for the routine detection of the carcinogen acrylamide in food, via rapid derivatisation pre-analysis. Anal. Chim. Acta 2008, 608 (2), 178-185.

Wu, J.; Shen, Y. D.; Lei, H. T.; Sun, Y. M.; Yang, J. Y.; Xiao, Z. L.; Wang, H.; Xu, Z. L., Hapten Synthesis and Development of a Competitive Indirect Enzyme-Linked Immunosorbent Assay for Acrylamide in Food Samples. Journal of Agricultural and Food Chemistry 2014, 62 (29), 7078-7084.

Luo, L.; Lei, H.-T.; Yang, J.-Y.; Liu, G.-L.; Sun, Y.-M.; Bai, W.-D.; Wang, H.; Shen, Y.-D.; Chen, S.; Xu, Z.-L., Development of an indirect ELISA for the determination of ethyl carbamate in Chinese rice wine. Anal. Chim. Acta 2017, 950, 162-169.

Diblikova, I.; Cooper, K. M.; Kennedy, D. G.; Franek, M., Monoclonal antibody-based ELISA for the quantification of nitrofuran metabolite 3-amino-2-oxazolidinone in tissues using a simplified sample preparation. Anal. Chim. Acta 2005, 540 (2), 285-292.

Kane, M. M.; Banks, J. N. Raising Antibodies. In Immunoassays: A Practical Approach; Gosling, J. P., Ed.; Oxford University Press: Oxford, United Kingdom, 2000; pp 37−50.
